# Fetal arrhythmias: prenatal evaluation and intrauterine therapeutics

**DOI:** 10.1186/s13052-020-0785-9

**Published:** 2020-02-12

**Authors:** Shi-Min Yuan, Zhi-Yang Xu

**Affiliations:** Department of Cardiothoracic Surgery, The First Hospital of Putian, Teaching Hospital, Fujian Medical University, 389 Longdejing Street, Chengxiang District, Putian, 351100 Fujian Province People’s Republic of China

**Keywords:** Arrhythmias, Fetus, Treatment

## Abstract

**Introduction:**

Fetal arrhythmias are a common phenomenon with rather complicated etiologies. Debates remain regarding prenatal diagnosis and treatment of fetal arrhythmias.

**Methods:**

The literature reporting on prenatal diagnosis and treatment of fetal arrhythmias published in the recent two decades were retrieved, collected and analyzed.

**Results:**

Both fetal magnetocardiogram and electrocardiogram provide information of cardiac time intervals, including the QRS and QT durations. M-mode ultrasound detects the AV and VA intervals, fetal heart rate, and AV conduction. By using Doppler ultrasound, simultaneous recording of the atrial and ventricular waves can be obtained. Benign fetal arrhythmias, including premature contractions and sinus tachycardia, do not need any treatment before and after birth. Sustained fetal arrhythmias that predispose to the occurrence of hydrops fetalis, cardiac dysfunction or eventual fetal demise require active treatments. Intrauterine therapy of fetal tachyarrhythmias has been carried out by the transplacental route. If maternal transplacental treatment fails, intraumbilical, intraperitoneal, or direct fetal intramuscular injection of antiarrhythmic agents can be attempted.

**Conclusions:**

The outcomes of intrauterine therapy of fetal tachyarrhythmias depend on the types or etiology of fetal arrhythmias and fetal conditions. Most are curable to a transplacental treatment by the first-line antiarrhythmic agents. Fetal cardiac pacings are effective methods to restore sinus rhythm in drug-resistant or hemodynamically compromised cases. Immediate postnatal pacemaker implantation is warranted in refractory cases.

## Introduction

Fetal arrhythmias are diagnosed in 1–3% of pregnancies [1], and account for 10–20% of the referrals to fetal cardiology [2]. In a non-randomized prospective study on 100 fetuses at 15–40 weeks of gestation for cardiac referal, 45 fetuses had cardiac arrhythmias, including premature atrial contractions (PACs) (28/45, 62.2%), atrial bigeminal ectopic beats (3/45, 6.7%), premature ventricular contractions (PVCs) (2, 4.4%), supraventricular tachycardia (SVT) (5/45, 11.1%), ventricular tachycardia (1, 2.2%), second-degree atrioventricular (AV) block (1, 2.2%) and complete AV block (5/45, 11.1%) [3]. A 10-year observational study on the pregnant women demonstrated 29 cases of fetal arrhythmias: 12 (41.4%) of which were fetal tachycardias (10 cases with SVT, 2 cases with atrial flutter (AF)), 5 (17.2%) were fetal bradyarrhythmias (all 5 cases with AV block), and 12 (41.4%) were fetal irregular cardiac rhythms (premature atrial beats) [4]. The overall incidence of malignant fetal arrhythmias, such as complete AV block and SVT, are relatively rare, found in 1:5000 pregnancies [5].

Genetic studies have shown that *GATA4*, *NKX2-5*, *TBX3*, and *TBX5* genes are responsible for cardiac structural development, whereas mutations of these genes may lead to congenital heart diseases and conduction disorders [6]. The occurrence of paroxysmal AF can be a result of *TBX5* gain-of-function mutations and overexpressions of *Nppa*, *Cx40*, *Kcnj2* and *Tbx3* genes [7]. *Na*_*v*_*1.5* gain-of-function mutation is proved to be associated with an increased risk of multifocal atrial and ventricular ectopies and dilated cardiomyopathy [8].

The fetuses with benign arrhythmias, such as PACs < 11 beats per minute (bpm) and sinusal tachycardias, did not need any treatment before or after birth, whereas those with postnatal arrhythmias associated with hemodynamic fluctuations require interventions, as they may lead to preterm delivery in some occasions [9]. Besides, sustained fetal arrhythmias predispose to the occurrence of hydrops fetalis, cardiac dysfunction, or even fetal demise [10]. Therefore, prenatal treatment is warranted for improving the fetal survival rate. The aim of the present study is to discuss the complex and challenging issue concerning the prenatal evaluation and intrauterine therapeutics of fetal arrhythmias.

### Diagnosis

M-mode ultrasound can detect the AV and ventriculoatrial (VA) intervals, fetal heart rate, AV conduction, and even ejection fraction [11], but detection qualities may be compromised by early detection in first trimester, unfavorable fetal position, hydrops fetalis, fetuses with cardiac contractile dysfunction and obese pregnant women [12]. Crowley et al. [13] reported that they used a two-dimensional scan head with M-mode recordings for the diagnosis of fetal arrhythmias. Fetal heart rate and rhythm were measured by detecting semilunar and AV valve opening and closing points, A waves, plus ventricular wall motion. In 2 fetuses of their patient setting, the arrhythmias were diagnosed using two-dimensional echo alone. The anatomic M-mode provides simultaneous two-dimensional real-time images and therfore can obtain good quality tracings of atria and ventricles than by standard M-mode views.

By using Doppler ultrasound, simultaneous recordings of the atrial and ventricular waves can be obtained. The mechanisms of SVT can be classified as mechanical VA intervals as short VA or long VA [14]. Measurement of the VA interval by Doppler echocardiography helps distinguish short VA interval from long VA interval types of fetal tachycardias, such as AV nodal reentrant tachycardia and permanent junctional reciprocating tachycardia [15].

The Doppler ultrasound records ascending aorta and superior vena cava flow velocity waveforms better than the M-mode. In fetuses with short VA tachycardia, it may display a distinctive Doppler flow velocity pattern with a 1:1 AV conduction and a tall A wave superimposed on the aortic ejection wave. It was regarded as a reentrant tachycardia through a fast-conducting AV accessory pathway. In long VA tachycardia, an A wave of normal amplitude with normal AV time interval could be detected in front of the aortic ejection wave [16]. Doppler waveforms detected from the inferior vena cava and the descending aorta helps in obtaining information of atrial and ventricular systoles simultaneously. However, this results may be compromised when the fetus is in an improper position for simultaneous recordings [17]. By detecting flow imaging frequency spectrum of the pulmonary arteries and pulmonary veins, the pulse Doppler echocardiography can determine the rhythm changes between the spectra and the arrhythmic patterns. This technique can readily identify atrial and ventricular systoles, and measure the PR interval [17].

Fetal electrocardiography (ECG) does not provide beat-to-beat analysis by detecting the signal averaging of electrocardiographic complexes. Thus, it is not helpful in diagnosing fetal rhythm and conduction disorders with irregular heart rates.

Fetal magnetocardiography (MCG) allows real-time detection and classification of arrhythmias [18] with better signal quality than electrocardiography due to more favorable transmission properties of the magnetic signals. It can be helpful in making prenatal diagnoses of a variety of fetal arrhythmias, such as complete AV block, premature contractions, paroxysmal SVT and Wolff-Parkinson-White syndrome and long QT syndrome [19]. However, the use of the magnetic analogue of ECG requires a magnetically shielded room. Both MCG and ECG may provide useful information on cardiac time intervals, such as the QRS and QT durations.

## Treatment

### Irregular arrhythmias

The majority of fetal arrhythmias are premature contractions. Capuruço et al. [9] reported that PACs were the most common fetal arrhythmias representing 55.5% (100/180), followed by bi- or trigemy (12/180, 0.7%), sinus tachycardia (18.3%, 33/180), SVT (15.6%, 28/180), and AF 0.4% (7/180). Most of the PACs are benign, and do not have a genetic cause, while a few PACs can be associated with congenital heart defects or as a manifestation of Costello syndrome caused by *HRAS* mutations [20]. In fetuses with premature contractions, fetal echocardiogram is useful for cardiac structural and functional assessments, and for disclosing the mechanisms of fetal isolated PACs and multiple ectopic beats [21]. PACs are usually benign and often resolve spontaneously, but follow-up is necessary for preventing from developing into ventricular tachycardia [22]. Respondek et al. [23] reported that PACs required antiarrhythmic treatments with digoxin, verapamil, or both in 14% of the cases.

Fetal PVCs were less common than PACs. Most isolated fetal PVCs usually resolve spontaneously. The sustained PVCs may also resolve within 6 weeks, and do not cause severe arrhythmias [24]. Fetal PVCs warrant close monitoring as they may develop into proxysmal ventricular tachycardias (VTs).

### Tachyarrhythmias

The transplacental administration of antiarrhythmic agents, including digoxin, flecainide, sotalol, and amiodarone, is applied for fetal tachycardia in many centers [25]. Flecanide and sotalol cross the placental barrier easier, especially in hydropic fetuses, and a higher drug concentration can be achieved in the amniotic fluid. The management protocols are shown in Table 1.

**Table 1:** The treatment protocol of fetal tachyarrhythmias [26]

Tachyarrhythmia	Short VA SVT/AF, nonhydropic	Short VA SVT/AF, hydropic	Long VA SVT
First-line	Digoxin	Digoxin and sotalol	Sotalol
Second-line	Digoxin and sotalol	Digoxin and flecainide/amiodarone	Flecainide
Third-line	Digoxin and flecainide		

*AF* atrial flutter, *SVT* supraventricular tachycardia, *VA* ventrioatrial conduction

The modes of administration, intraumbilical, intraamniotic, intraperitoneal, intramuscular and intracardiac, have been selected as routes of administration. The intraumbilical and intracardiac injections aim at a quick response to therapy by a direct access to the fetal circulation, but they pose a traumatic risk to the fetus. Intraperitoneal, intraamniotic, and intramuscular injections allow instant delivery of the drugs while the fetuses carry less traumatic injuries [27]. If maternal transplacental treatment fails, direct administrations, such as intraumbilical, intraperitoneal, or intramuscular injection of antiarrhythmic agents can be considered as alternative approaches. Intraumbilical administration of antiarrhythmic agents can be performed under ultrasound guidance, but with somewhat technical difficulty, especially when the fetus is in an unfavorable location. This direct treatment is indicated in cases of tachyarrhythmia with hydrops fetalis as an adjunctive to the higher dose of maternal transplacental therapy [28].

Fetal tachyarrhythmias are usually SVT (63.4%), AF (28.0%) and VT (8.5%). There are other rare types of fetal arrhythmias, such as ventricular tachycardia, junctional tachycardia, and multiforcal atrial tachycardia [14]. Fetal MCG may reveal a strong association between AF and an accessory pathway [29]. SVT mechanism was classified by mechanical VA time intervals as short VA or long VA. It has been reported that short VA interval occurred in 67 fetuses (80%) and long VA in 17 (20%). Treatment success was defined as conversion to sinus rhythm, or rate control, defined as > 15% rate reduction [14]. Digoxin, flecainide and sotalol can be the first-line treatments. Amiodarone, propafenone, and combined therapies are reserved for refractory fetal tachycardias [30].

Digoxin has been considered the first-line agent for the treatment of fetal SVT. Digoxin is praised for its safety and efficacy, but maternal higher doses are required to maintain a therapeutic serum level especially in the presence of hydrops fetalis [31]. Digoxin monotherapy showed a lower effective rate than combined digoxin and flecainide/sotalol for the treatment of fetal tachycardias (27.8% vs. 72.2%). The transplacental administration of combined digoxin and flecainide is an effective regimen for SVT with long VA interval [32].

Flecainide is an effective first-line treatment for fetal SVT with a high successful rate of 88.2%, low side effect and relatively easy utilization [33]. Flecainide is highly effective in achieving sinus rhythm in hydropic and nonhydropic fetuses with SVT, refractory SVT or SVT with signs of heart failure. Oral flecainide (100 mg three times daily) is reserved for those cases unresponsive to sotalol and digoxin [34]. It is more effective than digoxin, especially for hydropic fetal tachycardia, with no adverse fetal outcomes found [14]. Flecainide was preferred in converting SVT to normal sinus rhythm or in slowing AF to well-tolerated ventricular rates [35]. The conversion rate to sinus rhythm of flecainide for short VA SVT was higher than digoxin (96% vs. 69%, *P* = 0.01). For long VA SVT, the conversion rate to sinus rhythm did not differ significantly between the two drugs (67% vs. 50%, *P* = 0.13). In nonhydropic fetuses, the successful rate of flecainide was higher than digoxin (96% vs. 79%, *P* = 0.10). In hydropic cases, a same trend was observed (86% vs. 38%, *P* = 0.07 for flecainide vs. digoxin), while the successful rate of combined flecainide with amiodarone was 100%. The intrauterine or neonatal mortality rate in hydropic fetuses treated with flecainide was much lower than that treated with digoxin (0% vs. 43%, *P* = 0.06). Strizek et al. [36] reported that the successful rate was 81.2% (26/32) when treated with flecainide as a first-line therapy. The median time to conversion to sinus rhythm was 3 days (range 1–7 days) with flecainide monotherapy and 11.5 days (range 3–14 days) with a combined therapy. For AF persisting for 5 days, flecainide use achieved a much better heart rate control than soltalol [35]. With combined flecainide and digoxin therapy, conversion to sinus rhythm occurred within 5 days (range, 0–14 days). Most fetuses (75%) converted to sinus rhythm within 7 days of treatment [37].

Sotalol is usually well-tolerated and has little or no negative inotropic effect on the fetal heart. It should be used with small doses cross the placenta [31]. Sotalol is the best treatment for fetal AF in most cases and is a safe and effective therapy for SVT [35]. Rebelo et al. [38] reported that successful drug treatment with sotalol in 5/6 (83.3%) cases with no adverse effects for the mothers. In comparison to flecainide or digoxin, sotalol was less effective to convert SVT to sinus rhythm. Shah et al. [39] documented response to sotalol (43%) or sotalol/digoxin (57%) as first-line treatment in 21 pregnancies. The time to conversion to sinus rhythm for sotalol varied from 1 to 5 days (median 1 day) for Shah et al. [39], 1–35 days (median 7.5 days) for van der Heijden et al. [40] and a median of 12 days for Jaeggi et al. [41] Freedom from arrhythmia on maintenance therapy was 93 and 90% at 1 and 3 months, respectively. The overall mortality was 8%, only 4% of which was arrhythmia-related. In the absence of hydrops, fetal AF/SVT was associated with low morbidity and mortality rates.

Amiodarone is a second-line treatment, especially in hydropic fetuses with SVT [27]. In the event of life-threatening fetal arrhythmia, direct fetal therapy with adenosine and amiodarone can be a last resort [34]. In cases of refractory SVT with severe hydrops fetalis, the treatment regimen can be a maternal oral loading dose of 200 mg, followed by fetal intraperitoneal dose of 4–7 mg/kg. Fetal intraperitoneal amiodarone was successful in 75% (6/8) cases. The frequency of intraperitoneal injections depended on the therapeutic response, usually 1–4 doses, but up to 11 doses in an extreme case with a conversion time of 11.5 days after the initial injection. Besides, immediate cardioversion was also observed in a fetus receiving intraumbilical injection of amiodarone. Hydrops fetalis resolved in 62.5% (5/8) fetuses, with a mean resolution time of 28.4 days [42].

In general, digoxin is widely accepted as a first-line antiarrhythmic drug. Sotalol, flecainide and amiodarone are used as second-line drugs when digoxin fails to achieve conversion to sinus rhythm. For fetuses with hydrops and fetal SVT with long VA interval, digoxin is rarely effective. For fetuses with hydrops, the placental transfer of the digoxin is limited. Sotalol and flecainide have good placental transfer ability, and they should be used as first-line treatment for hydropic fetal tachyrrhythmias. Fetal direct intramuscular injection of digoxin with maternal amiodarone use is an effective alternative. The treatment of choices for fetal tachyarrhythmias was listed in Table 2.

**Table 2:** The treatment of choices for fetal tachyarrhythmias [43, 44]

Parameter	Digoxin	Flecainide	Sotalol	Amiodarone
Indication	Paroxymal SVT, short VA SVT, nonhydropic fetuses	SVT with NIHF, refractory SVT, SVT with heart failure unresponsive to soltalol and digoxin	AF, SVT	SVT resistant to digoxin, AF
Dose	Loading: 1.5–2 mg over 24–48 h; Maintenance: 0.375–1 mg/day	Loading: 200–300 mg divided b.i.d., or t.i.d.; Maintenance: 450 mg/day if no response	Loading: 160–320 mg divided b.i.d.; Maintenance: increased to 480 mg/day	Loading: 1600–2400 mg/day 2–4 times per day; Maintenance: 200–400 mg/day b.i.d.
Route	p.o., or i.v.	p.o.	p.o.	p.o., or i.v.
Fetal/maternal serum level (%)	40–90			10–50
Advantage	Safe and effective		Not accumulate in fetus, not cause intrauterine growth retardation	Little or no negative inotropic effect
Adverse effect	Digoxin monotherapy showed a lower effective rate than combined; Hydropic fetuses refractory to digoxin	Intrauterine death	Negative inotropic effect, intrauterine death	Arrhythmogenic effect, fetal thyroid functional impairement, maternal thrombocytopenia and skin rash

*AF* atrial flutter, *NIHF* nonimmune hydrops fetalis, *SVT* supraventricular tachycardia, *VA* ventrioatrial conduction

Stirnemann et al. [45] applied fetal esophageal pacing with a bipolar pacing esophageal lead (FIAB Esokid 4S, Firenze, Italy) positioned behind the left atrium for the treatment of fetal AF. The lead was connected to an asynchronous esophageal pacemaker. It showed an immediate conversion to sinus rhythm.

### Bradyarrhythmias

Fetal bradycardias may be due to sinus bradycardia, blocked PACs, or high degree AV block [46]. Transient bradycardia is somewhat common in the developing fetus and is usually benign. Complete AV block occurred in 2.6% of fetuses with irregular cardiac rhythyms [47]. Capuruço et al. [7] reported that the prevalence of fetal bradyarrhythmias was 3.4% (62/1821). Fetal bradycardias may occur in the presence of fetal hypoxia [48], associated congenital structural disorders [49], maternal connective tissue disorders [50], positivity of maternal SSA/Ro and/or SSB/La autoantibodies [50], or due to an unknown cause [51]. Sinus bradycardias are often caused by fetal hypoxia or immaturity of the cardiac conduction system. The transient fetal bradycardia is benign and often need no fetal treatment. The two most common congenital heart defects associated with AV block are left atrial isomerism and discordant AV connection. Maternal anti-SSA/SSB antibody positivity is another cause of fetal AV block. Long QT syndrome can cause 2:1 AV block or sinus bradycardia. Blocked atrial bigeminy also resembles 2:1 AV block and causes fetal bradycardia. Both M-mode and Doppler echocardiography can help diagnose sinus bradycardia. The prolonged episodes of sinus bradycardia can be caused by fetal distress as a result of fetal hypoxia and acidosis, long QT syndrome, and congenital sinus node dysfunction [34]. Fetal bradycardia with either congenital heart defects or fetal hydrops significantly worsens their prognoses. Moreover, heart function and congenital heart defects exaggerate the severity of congestive heart failure [15].

Fetal complete AV block with structural heart disease often shows a worse prognosis, such as fetal demise or pacemaker implant requirement. All those with complete AV block by maternal autoantibodies positivity survived, but 42.8% needed a pacemaker. The high risks of perinatal demise was often associated with fetal hydrops, structural defects, poor ventricular function and HR < 55 bpm. Regular screening by fetal echocardiography and transplacental treatment could prevent this risk factor [9].

Miyoshi et al. [52] analyzed 29 cases of fetal bradycardia with structural heart disease, including isomerism (*n* = 22), corrected transposition of the great arteries (*n* = 4), and critical pulmonary stenosis (*n* = 3). The mechanisms of fetal bradycardia were complete AV block (14/29, 48.3%), second-degree AV block (8/19, 42.1%). Besides, 16 (84.2%) cases had sick sinus syndrome. Fetal demise occurred in 5 (26.3%), and neonatal death in 10 (41.7%). The neonatal and overall survival rates for fetal bradyarrhythmia with structural heart disease were much higher, which were 66 and 48%, respectively. Pacemaker implantation was warranted in 17 (89.5%) cases. In utero β-stimulants were used in 13 (68.4%) cases and effective in 6 (31.6%). The fetuses with corrected transposition of the great arteries or ventricular rate ≥ 70 bpm had a better survival rate. A ventricular rate < 55 bpm, fetal cardiac dysfunction and hydrops fetalis (*P* = 0.04) were significant predictive risk factors of a higher mortality rate.

β-stimulants, such as ritodrine, terbutaline, and salbutamol, and steroids have been reported to be effective transplacental treatments for fetal AV block, and they may increase fetal ventricular rate by 10–20% and reverse hydrops as well. Transplacental administration of steroids, such as dexamethasone and betamethasone, are effective for fetal AV block caused by positive maternal autoantibodies. Transplacental administration of steroids is also effective for the treatment of myocarditis, and improves fetal cardiac function. Early delivery and direct ventricular pacing is a reasonable option when the fetal heart rate decreases progressively and hydrops fetalis develops in the presence of fetal AV block [15].

In 1986, Carpenter et al. [53] reported, for fetuses with complete AV block with poor responses to transplacental therapies, fetal transthoracic ventricular pacing ensures temporary fetal ventricular rate acceleration. It was worthwhile mentioning that the initial ventricular pacing threshold was very low in the hydropic fetus. In 1994, Waikimshaw et al. [54] described percutaneous transvenous intracardiac cardiac pacing performed in a case of fetal AV block via the fetal umbilical vein under ultrasound guidance. After the pacing wire was advanced into the right atrium and subsequently the right ventricle, the pacing rate was set up at 140 bpm. It is indicated for fetal long QT syndrome type 2 and complete AV block [45].

## Prognosis

Fetal arrhythmia has various types and different prognosis. Individualized treatment and clinical treatment should be determined according to specific types. Premature contractions are the most common type of fetal arrhythmia, and the prognosis is good in the near and long terms, and fetal growth and development are not affected [55]. The prevalence of rapid fetal arrhythmia, especially SVT, is relatively high, accounting for 0.4–0.6% of all fetuses. Most of the rapid fetal arrhythmia is a nonorganic lesion, mostly transient. Fetal bradycardia has shown limited therapeutic efficacy, and early treatment with steroids and/or plasmapheresis remains controversial. The clinical outcome and prognosis of patients are usually determined by the type and extent of cardiac malformation [55]. Therefore, when fetal arrhythmia, in particular fetal bradycardia, is found, special attention should be paid to whether cardiac structural abnormalities is present [55]. Appropriate clinical measures should be taken into consideration with regard to outcomes and prognosis.

## Conclusions

Benign fetal arrhythmias, such as premature contractions and sinus tachycardia, do not need any perinatal treatments. Sustained fetal arrhythmias that predispose to the occurrence of hydrops fetalis, cardiac dysfunction, or even fetal demise require early treatments. The effect of intrauterine therapy of fetal tachyarrhythmias depends on the types or etiology of fetal arrhythmia and fetal conditions (hydrops fetalis, cardiac function, and maternal autoantiboy positivity, etc.). to the conversion rate was high with the use of the first-line antiarrhythmic agents via the transplacental route. Fetal cardiac pacings are effective methods to restore sinus rhythm in drug-resistant or hemodynamically compromised cases. Immediate postnatal pacemaker implantation is warranted in refractory cases.

## Data Availability

Based on literature review.
